# Association between lncRNAs in plasma exosomes and diabetic retinopathy

**DOI:** 10.3389/fendo.2022.987488

**Published:** 2022-09-14

**Authors:** Qingqing Ye, Lian Li, Zhoujie Shao, Miao Xu, Li Li, Qianqian Yan, Bin Huang, Tian Zhao

**Affiliations:** ^1^ Clinical Laboratory, Beilun District People’s Hospital, Ningbo, China; ^2^ Department of Prevention and Control, Ningbo Kangning Hospital, Ningbo, China; ^3^ Department of Endocrinology, Ningbo First hospital, Ningbo, China; ^4^ Department of Global Health, Ningbo Institute of Life and Health Industry, University of Chinese Academy of Sciences, Ningbo, China; ^5^ Department of Emergency Medicine, Beilun District People’s Hospital, Ningbo, China

**Keywords:** type 2 diabetes mellitus, diabetic retinopathy, exosome, lncRNA, case–control study

## Abstract

**Background:**

Long noncoding RNA (lncRNA) in plasma exosomes is a potential non-invasive diagnostic biomarker for diabetic retinopathy (DR). However, the changes in plasma exosomal lncRNAs and diagnostic relevance in patients with DR patients remain unclear.

**Methods:**

A case–control study with type 2 diabetes mellitus (T2DM) and patients with comorbid DR were enrolled, and their clinical information and blood samples were collected. Plasma exosomes were extracted, and the relative expression levels of representative differentially expressed exosomal lncRNAs were determined. A logistic regression model was used to analyze the relationships of DR with relative lncRNA expression and DR-related factors, and receiver operating characteristic (ROC) curve analysis was used to evaluate the value of exosomal lncRNAs for DR diagnosis.

**Results:**

Sixty-two patients with T2DM and sixty-two patients with DR were matched by age, sex, and disease duration. The fasting blood glucose concentration, glycosylated hemoglobin level (HbA_1c_), and relative expression of the plasma exosomal lncRNA *DLX6-AS1* were significantly higher in the DR group than in the T2DM group, whereas the 2-h C-peptide concentration and relative expression of the lncRNAs *PRINS* and *FAM190A-3* were lower in the DR group. After adjusting for relevant confounders, the fasting blood glucose concentration, HbA_1c_ level, 2-h C-peptide concentration, and relative expression of lncRNA *DLX6-AS1*, *PRINS*, and *FAM190A-3* were found to be associated with DR. Both *DLX6-AS1* [area under the curve (AUC): 0.658 (0.562–0.754)], *PRINS* [AUC: 0.798 (0.722–0.873)], and *FAM190A-3* [AUC: 0.603 (0.503-0.702)] expression had predictive value for DR diagnosis. The combination of *DLX6-AS1* and *PRINS* yielded an AUC of 0.813 (0.740–0.886). In males, the combination of *DLX6-AS1* and *PRINS* yielded an AUC of 0.860 (0.780–0.940).

**Conclusion:**

The fasting blood glucose concentration, HbA_1c_ level, and exosomal *DLX6-AS1* expression were identified as risk factors for DR, whereas the 2-h C-peptide concentration and exosomal *PRINS* and *FAM190A-3* were identified as protective against DR. The combination of exosomal *DLX6-AS1* and *PRINS* had good diagnostic value for DR in the general population and males. More attention should be paid to the role of exosomal *PRINS* expression as a predictive and diagnostic DR biomarker in females.

## 1 Introduction

Type 2 diabetes mellitus (T2DM) is a chronic metabolic disease characterized by insulin resistance and elevated blood glucose concentration. In 2019, the population of people with diabetes mellitus reached 463 million worldwide, and it is expected to expand to 629 million by 2045 (1). More than 90% of these cases of diabetes involve T2DM, which has become one of the most important global public health problems (2). Diabetic retinopathy (DR) is a common and serious ocular microvascular complication of diabetes mellitus, and more than 30% of patients with diabetes will develop this complication. Worldwide, approximately 93 million people are affected by DR, which has become the main cause of vision loss and even blindness among working-age people (3, 4). Currently, DR is treated mainly at an advanced stage, when the patient’s vision has been severely damaged. The treatment for DR is severely invasive and always accompanied by various complications (4). Therefore, a highly sensitive, non-invasive biomarker that could be used to screen for DR in people with diabetes mellitus and enable timely and early intervention would be significant in terms of public health efforts to prevent and treat DR (5).

Exosomes are extracellular vesicles containing specific proteins, lipids, functional mRNA, non-coding RNA and other biologically active substances. Some studies have found that in humans, changes in the number and composition of exosomes might reflect the development and pathological status of DR. Long noncoding RNAs (lncRNAs) are non-protein-encoding transcripts longer than 200 nucleotides that plays an important role in transcriptional (6), chromatin (7), and post-transcriptional regulation (8). Especially lncRNAs localized in exosomes can be secreted and enter recipient cells, and are involved in epigenetics, cell type reprogramming and genome instability regulation (9). LncRNAs *MALAT1* (10), *ANRIL* (11, 12), *RNCR3* (13), *MIAT* (14), and *Sox2OT* (15) have been reported to be related to the pathological changes of DR. However, most studies have focused on changes in lncRNAs in the serum or plasma of patients with DR. It remains unclear whether the level of lncRNA expression in plasma exosomes has potential diagnostic value for DR.

This study aimed to compare the relative expression of lncRNAs in plasma exosomes between patients with T2DM with and without DR, and to explore the correlations of DR with the expression of plasma exosomal lncRNAs and the presence of some clinical indicators. Our study has provided evidence elucidating the role of exosomes in the pathogenesis of DR and, potentially, in early screening for this condition.

## 2 Materials and methods

### 2.1 Study design

A case–control study with a 1:1 paired design was conducted in Ningbo First Hospital, China. Patients with comorbid T2DM and DR who were hospitalized between September 2018 and September 2019 were enrolled as the case group. Patients with T2DM only who were hospitalized during the same period were identified as the control group and matched with cases by age ( ± 3 years), sex, and disease duration ( ± 5 years) to eliminate confounding factors. Finally, 62 cases and 62 controls were included in the study.

Relevant demographic data were collected from all participants using a unified questionnaire (**Supplementary Table 1**), and the results of physical examinations and routine biochemical tests were collected through the medical record system. A 10-mL sample of venous blood was drawn from each patient within 24 hours of hospital admission, and placed in the ethylene diamine tetraacetic acid anticoagulant tube. 124 blood samples were obtained, and plasma was extracted and stored in a freezer at -80°C. An average of 4-5 mL plasma sample was collected from each patient. This study was conducted in strict accordance with the principles of the internationally accepted Declaration of Helsinki and was approved by the Ethics Committee of Ningbo First Hospital before initiation (approval number: 2017-R048). All participants received an explanation of the background and content of the project from experienced physicians, and all voluntarily provided signed informed consent.

### 2.2 Study definition

The inclusion criteria for the case group (DR) were a diagnosis of T2DM and clearly observable retinal hemorrhage, retinal microaneurysm, vitreous hemorrhage, or retinal neovascularization during a fundus examination with mydriasis conducted by an experienced ophthalmologist. The inclusion criteria for the control group were a diagnosis of T2DM and no abnormalities in a fundus examination after mydriasis. The diagnosis of T2DM was based on the diagnostic criteria issued by the American Diabetes Association (16).

The exclusion criteria for all potential participants were diagnosis with other types of diabetes; other complications of diabetes; cardiac insufficiency; severe malignancy; poor blood pressure control; severe liver and kidney dysfunction; pregnancy or lactation; acute infection; other eye diseases.

### 2.3 Laboratory procedures

#### 2.3.1 Exosome extraction and identification

5 patients were randomly selected from each group, and exosome was extracted from 2-mL plasma sample using ExoQuick exosome precipitation solution (EXOQ5A-1, SBI, USA). Western blotting was performed using antibodies specific for the representative exosome marker proteins CD63 and CD9, and an antibody specific for calnexin (ab59479; ab223052; ab112995, Abcam, UK) as a negative control. The morphological characteristics of exosomes were observed using a transmission electron microscope (TecnaiG2, FEI, USA), and the exosome particle sizes were determined (Nanosight LM10, FEI, USA) to identify plasma exosomes.

##### 2.3.2 Exosome sequencing and analysis

Total RNA was extracted from plasma exosomes using QIAGEN miRNeasy Micr Kit (217084, QIAGEN, Germany). After RNA fragmentation, random primer reverse transcription, dUTP incorporation, adenosine tailing with A, adapter ligation, dUTP strand degradation, polymerase chain reaction (PCR), RNA-Seq libraries were constructed using KAPA RNA HyperPrep Kit Illumina^®^ (Illumina, USA) and sequenced using HiSeq X10 PE150 mode (Illumina, USA). The rRNA sequences were removed and the effective reads were mapped with the reference genome. After obtaining transcriptome assembly, lncRNAs expression were detected. Difference analysis was performed by using R version 3.6.1 (http://www.r-project.org/). A heat map and volcano map were used to show differences in exosomal lncRNA expression between groups, which enrich in Kyoto Encyclopedia of Genes and Genomes (KEGG) pathway.

##### 2.3.3 Real-time quantitative PCR

Exosome was extracted from 250-µL plasma samples from each patient. The total RNA samples were reverse transcriptionized into cDNA (PrimeScript™ RT Reagent Kit with gDNA Eraser, Takara, Japan). Five lncRNAs were selected as candidate genes for further sample validation. Detailed information and sequences of these target lncRNAs were obtained from the LNCipedia website (http://www.lncipedia.org/), and relevant primers were designed **(** **Supplementary Table 2**). Real-time quantitative PCR was performed using cDNA as template to detect and verify the relative expression of candidate differential lncRNAs (TransStart^®^ Tip Green qPCR SuperMix, TRANS, China).

### 2.4 Statistical analysis

The t-test was used to compare normally distributed variables between the two groups, which were displayed as means ± standard deviations. The Mann–Whitney U test were used to compare not normally distributed variables between the two groups, which were described as the median (interquartile range).The chi-square test was used to compare categorical variables between the two groups. A binary logistic regression model, including univariate and multivariate analyses, was used to analyze the relationships between DR and the expression levels of exosomal lncRNAs and other biochemical factors. Pearson correlation coefficient was used to analyze the correlation between exosomal lncRNAs and biochemical factors. Receiver operating characteristic (ROC) curve analysis was used to evaluate the predictive value of exosomal lncRNA expression for DR diagnosis. SPSS 24.0 (IBM Corp., Armonk, USA) was used to analyze the data, and a two-sided P value < 0.05 was regarded as statistically significant.The 100KB upstream and downstream coding genes of lncRNAs were searched *via* cis, a non-coding RNA regulation of transcriptional activation and expression of adjacent mRNAs (17). GeneOntology (GO) analysis was performed using R 3.6.1. GO terms with P < 0.05 were considered to be significantly enriched, and were visualized with the ggplot 2 package and Cytoscape 3.9.1 (http://www.cytoscape.org/).

## 3 Results

### 3.1 Demographic and clinical characteristics of patients in the DR and T2DM groups

The demographic and clinical characteristics of the participants are shown in **Table 1**. The waist-to-hip ratio was higher in the DR group than in the T2DM group (0.95 ± 0.07 vs. 0.92 ± 0.06, *P* = 0.030). The total bilirubin (16.20 ± 6.33 vs. 13.40 ± 5.54 μmol/L, *P* = 0.029) and uric acid (322.98 ± 90.75 vs. 283.74 ± 82.86 mmol/L, *P* = 0.040) were significantly higher in the T2DM group than in the DR group.

**Table 1:** Demographic and clinical characteristics of patients in DR and T2DM groups.

	DR (n = 62)	T2DM (n = 62)	*t*/χ^2^/*Z*	*P*
**Demographic characteristics**				
Age (years)	56.18 ± 10.86	55.42 ± 10.56	-0.394	0.694
Sex (males/females)	39/23	39/23	–	–
Body mass index (kg/m^2^)	25.35 ± 5.52	24.97 ± 3.14	-0.473	0.637
Waist-to-hip ratio	0.95 ± 0.07	0.92 ± 0.06	-2.201	0.030
Diastolic blood pressure (mmHg)	79.48 ± 12.98	80.71 ± 12.63	0.534	0.594
Systolic blood pressure (mmHg)	137.07 ± 18.20	136.07 ± 20.48	-0.286	0.775
Marriage			0.226	0.635
Married	60	58		
Other	2	3		
Labor intensity			4.365	0.113
Mild	55	48		
Moderate	7	11		
Serve	0	3		
Smoker			4.439	0.109
Now	15	10		
Former	12	7		
None	32	45		
Alcohol consumption			1.025	0.599
Now	20	15		
Former	38	42		
None	4	5		
Hypertension (yes/no)	35/27	33/29	0.130	0.718
Hyperlipidemia (yes/no)	43/19	39/23	0.576	0.448
Fatty liver (yes/no)	52/10	44/18	2.952	0.086
**Biochemical indicators**				
Total cholesterol (mmol/L)	4.80 ± 1.13	4.96 ± 1.19	0.668	0.506
Low-density lipoprotein cholesterol (mmol/L)	3.10 ± 0.89	3.11 ± 1.05	0.060	0.952
High-density lipoprotein cholesterol (mmol/L)	1.14 ± 0.24	1.17 ± 0.18	0.795	0.429
Triglyceride (mmol/L)	1.74 ± 0.97	2.19 ± 3.26	0.866	0.389
Total bilirubin (μmol/L)	13.40 ± 5.54	16.20 ± 6.33	2.216	0.029
Uric acid (mmol/L)	283.74 ± 82.86	322.98 ± 90.75	2.091	0.040
Homocysteine (μmol/L)	9.68 ± 2.46	10.94 ± 4.28	1.065	0.294
Hemoglobin (g/L)	107.01 ± 61.97	106.72 ± 68.75	-0.019	0.985
Free fatty acid (mmol/L)	526.63 ± 223.76	535.71 ± 223.55	0.131	0.896
**T2DM related indicators**				
Duration of T2DM (years)	7.75 ± 4.91	6.96 ± 4.61	-0.924	0.179
Fasting blood glucose (mmol/L)	10.88 ± 3.23	8.26 ± 2.83	-3.457	0.001
2h blood glucose (mmol/L)	15.76 ± 5.36	13.72 ± 5.57	-1.478	0.145
Fasting insulin (mmol/L)	6.36 (3.73-10.07)	9.88 (6.15-17.12)	-1.720	0.085
2h insulin (mmol/L)	18.71 (11.37-28.20)	30.08 (17.83-63.43)	-2.430	0.015
Fasting C-peptide (nmol/mL)	1.94 ± 0.90	2.40 ± 0.87	2.075	0.042
2h C-peptide (nmol/mL)	3.82 ± 1.73	6.00 ± 3.00	3.611	0.001
Glycosylated hemoglobin (HbA_1c_, %)	9.19 ± 2.20	7.53 ± 1.39	-3.826	<0.001

DR, Diabetic retinopathy; T2DM, Type 2 diabetes mellitus.

Besides, some indicators closely related to T2DM, such as the fasting blood glucose concentration (10.88 ± 3.23 vs. 8.26 ± 2.83 mmol/L, *P* = 0.001) and glycosylated hemoglobin (HbA1c) level (9.19 ± 2.20 vs. 7.53 ± 1.39%, *P* < 0.001) were higher in the DR group than in the T2DM group. In contrast, the 2-h insulin [30.08 (17.83–63.43) vs. 18.71 (11.37–28.20) mmol/L, *P* = 0.015], fasting C-peptide (2.40 ± 0.87 vs. 1.94 ± 0.90 nmol/mL, *P* = 0.042), and 2-h C-peptide concentrations (6.00 ± 3.00 vs. 3.82 ± 1.73 nmol/mL, *P* = 0.001) were higher in the T2DM group than in the DR group.

### 3.2 The difference of plasma exosome lncRNA expression between the DR and T2DM groups

Next, the extracted plasma exosomes were analyzed. We observed the expression of CD63 and CD9 and the absence of calnexin in the exosomes (**Supplementary Figure 1A**), indicating that marker proteins, CD63 and CD9, only came from exosomes rather than cells. The particle sizes consistent with the known range of exosome sizes (50-150nm) (18) (**Supplementary Figure 1B**) and transmission electron microscopy revealed disc-like structures (**Supplementary Figure 1C**), suggesting that the extracts contained pure plasma exosomes.

The exosomes were subjected to sequence analysis to explore differences in the expression of exosomal lncRNAs between the DR and T2DM groups (**Supplementary Figure 2A**). We identified 130 lncRNAs genes that were upregulated and 134 that were downregulated compared DR to T2DM group (**Supplementary Figure 2B**). KEGG enrichment analysis of lncRNAs revealed that the functions of differential genes were reflected in maturity onset diabetes of the young and TGF-beta signaling pathway, which were closely related to our research (**Supplementary Figure 2C**). The lncRNAs distal-less homeobox 6 antisense 1 (*DLX6-AS1*), psoriasis-susceptibility-related RNA gene induced by stress (*PRINS*), family with sequence similarity 190, member A3 (*FAM190A-3*), aminoacylase-1 (*ACY1*), and Rho GTPase activating protein (*ARHGAP*) were selected as candidate genes for subsequent data analysis.

We found that, in the total study population, the relative expression of *DLX6-AS1* was higher (1.35 ± 0.74 vs. 1.04 ± 0.51, *P* = 0.008) in the DR group than in the T2DM group, whereas the relative expression of *PRINS* (0.99 ± 0.14 vs. 1.20 ± 0.25, *P* < 0.001) and *FAM190A-3* (1.04 ± 0.14 vs. 1.09 ± 0.13, *P* = 0.038) was lower in the DR group. However, a subgroup analysis by sex revealed that the difference in *DLX6-AS1* expression between the DR and T2DM groups was only significant in males (1.53 ± 0.84 vs. 1.07 ± 0.59, *P* = 0.007). The difference in *PRINS* expression between the DR and T2DM groups was significant in both males (0.98 ± 0.15 vs. 1.21 ± 0.25, *P* < 0.001) and females (0.99 ± 0.14 vs. 1.18 ± 0.25, *P* = 0.003), whereas no significant between-group difference in *FAM190A-3* was observed for either sex. (**Table 2**).

**Table 2:** The differences in expression levels of exosome lncRNA between DR and T2DM groups.

lncRNA	DR	T2DM	*t*	*P*
***DLX6-AS1***	1.35 ± 0.74	1.04 ± 0.51	-2.683	0.008
Males	1.53 ± 0.84	1.07 ± 0.59	-2.766	0.007
Females	1.02 ± 0.37	0.97 ± 0.32	-0.533	0.597
***PRINS***	0.99 ± 0.14	1.20 ± 0.25	5.757	<0.001
Males	0.98 ± 0.15	1.21 ± 0.25	4.770	<0.001
Females	0.99 ± 0.14	1.18 ± 0.25	3.170	0.003
***FAM190A-3***	1.04 ± 0.14	1.09 ± 0.13	2.099	0.038
Males	1.04 ± 0.17	1.09 ± 0.12	1.498	0.138
Females	1.05 ± 0.09	1.11 ± 0.15	1.519	0.136
***ACY1***	1.29 ± 0.54	1.36 ± 0.71	0.554	0.581
Males	1.32 ± 0.52	1.25 ± 0.41	-0.547	0.586
Females	1.25 ± 0.59	1.56 ± 1.05	1.140	0.261
***ARHGAP***	1.31 ± 0.52	1.28 ± 0.37	-0.290	0.772
Males	1.39 ± 0.61	1.22 ± 0.29	-1.406	0.164
Females	1.17 ± 0.25	1.39 ± 0.46	1.871	0.069

lncRNA, long noncoding RNA; DR, Diabetic retinopathy; T2DM, Type 2 diabetes mellitus.

Studies have shown that exosomes can mediate insulin sensitivity in patients with fatty liver (19). The pathogenesis of T2DM involves a change in insulin sensitivity, and a correlation between fatty liver and T2DM has been identified (20). Similarly, we found differences in exosomal gene expression between patients with and without fatty liver. Specifically, a significant increase in *DLX6-AS1* expression in the DR group relative to the T2DM group was only observed in patients without a history of fatty liver (1.37 ± 0.67 vs. 0.91 ± 0.27, *P* = 0.019). Significant reductions in the expression of *PRINS* (0.98 ± 0.15 vs. 1.21 ± 0.26, *P* < 0.001) and *FAM190A-3* (1.05 ± 0.07 vs. 1.09 ± 0.13, *P* = 0.038) in the DR group compared with the T2DM group were only observed in patients with fatty liver. There were no differences in the relative expression of *ACY1* and *ARHGAP* between the DR and T2DM groups, regardless of the fatty liver status (*P* > 0.05) (**Supplementary Table 3**).

### 3.3 Analysis of the predictive value of plasma exosome lncRNAs for DR diagnosis

We explored the factors influencing DR at the biochemical and exosome levels and identified significant differences between the two study groups. Univariate logistic regression analysis identified associations of the total bilirubin [0.922 (0.855–0.994)], uric acid [0.995 (0.990–1.000)], fasting blood glucose [1.321 (1.102–1.583)], fasting C-peptide [0.543 (0.296–0.997)], and 2-h C-peptide concentrations [0.664 (0.511–0.864)] and the HbA1c level [1.662 (1.223–2.258)] with DR. Additionally, the exosomal expression of *DLX6-AS1* [3.214 (1.519–6.800)], *PRINS* [0.167 (0.077–0.363)], and *FAM190A-3* [0.319 (0.014–0.729)] was found to be associated with DR. After adjusting for age, sex, and other possible confounders, the associations of the fasting blood glucose [1.402 (1.139–1.727)] and 2-h C-peptide concentrations [0.588 (0.420–0.824)], HbA1c level [2.021 (1.357– 3.011)], and the expression of *DLX6-AS1* [3.505 (1.505–8.163)], *PRINS* [0.130 (0.051–0.329)], and *FAM190A-3* [0.317 (0.125–0.802)] remained associated with DR. These findings imply that the fasting blood glucose concentration, HbA_1c_ level, and exosomal *DLX6-AS1* might be risk factors for DR, whereas the 2-h C-peptide concentration and exosomal *PRINS* and *FAM190A-3* might protect against DR (**Table 3**). Interestingly, the protective factor *PRINS* was negatively correlated with the risk factor HbA_1c_ level (r = -0.247, *P* = 0.034), and positively correlated with the protective factor 2-h C-peptide concentration (r = 0.265, *P* = 0.032), suggesting a potential effect of *PRINS* on HbA_1c_ and 2h C-peptide, although the correlation was weak (**Supplementary Table 4**).

**Table 3:** Univariate and multivariate logistic regression analysis of the association between DR and biochemical factors and exosome lncRNA.

	Univariate		Multivariate	
	OR (95% CI)	*P*	OR (95% CI)	*P*
Biochemical factor*				
Total bilirubin	0.922 (0.855-0.994)	0.034	0.944 (0.865-1.030)	0.195
Uric acid	0.995 (0.990-1.000)	0.044	0.995 (0.990-1.001)	0.088
Fasting blood glucose	1.321 (1.102-1.583)	0.003	1.402 (1.139-1.727)	0.001
2h insulin	0.989 (0.973-1.005)	0.179	0.993 (0.977-1.009)	0.387
Fasting C-peptide	0.543 (0.296-0.997)	0.049	0.597 (0.310-1.150)	0.123
2h C-peptide	0.664 (0.511-0.864)	0.002	0.588 (0.420-0.824)	0.002
HbA_1c_	1.662 (1.223-2.258)	0.001	2.021 (1.357-3.011)	0.001
Exosome lncRNA**				
*DLX6-AS1*	3.214 (1.519-6.800)	0.002	3.505 (1.505-8.163)	0.004
*PRINS*	0.167 (0.077-0.363)	<0.001	0.130 (0.051-0.329)	<0.001
*FAM190A-3*	0.319 (0.014-0.729)	0.007	0.317 (0.125-0.802)	0.015
*ACY1*	0.616 (0.283-1.337)	0.220	0.746 (0.315-1.763)	0.504
*ARHGAP*	1.448 (0.670-3.131)	0.346	1.230 (0.516-2.937)	0.640

DR, Diabetic retinopathy; lncRNA, long noncoding RNA; T2DM, Type 2 diabetes mellitus; Glycosylated hemoglobin, HbA_1c_.

*Adjusted for age, gender, body mass index, waist to hip ratio.

**Adjusted for age, gender, body mass index, waist to hip ratio, total bilirubin, glucose.

We found that exosomal *DLX6-AS1* was only associated with DR in participants who were male [6.688 (2.135–20.952], aged > 60 years [10.061 (1.473–68.719)], had a history of smoking [8.048 (1.259-51.457)], and/or consumed alcohol [12.942 (1.216–137.774)]. Exosomal *FAM190A-3* was only associated with DR in participants who were male [0.246 (0.075–0.799)], aged > 60 years [0.077 (0.011–0.522)], and did not consume alcohol [0.228 (0.072–0.717)]. In contrast, exosomal *PRINS* was associated with DR in all subgroups. We found no other interactions of sex, age, smoking history, alcohol consumption, or other variables with the associations between exosomal lncRNAs and DR (**Supplementary Table 5**).

**Figure 1:**
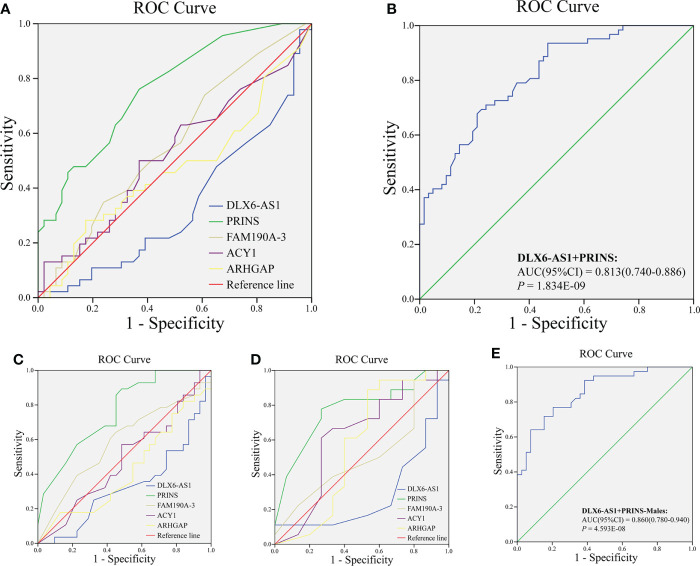
Analysis of diagnostic value of exosomal lncRNA in DR. The ROC curve of **(A)** lncRNA *DLX6-AS1*, *PRINS*, *FAM190A-3*, *ACY1*, and *ARHGAP*; **(B)** combining *DLX6-AS1* with *PRINS* to diagnose DR in all population. The ROC curve of lncRNA *DLX6-AS1*, *PRINS*, *FAM190A-3*, *ACY1*, and *ARHGAP* to diagnose DR in **(C)** males and **(D)** females. **(E)** The ROC curve of combining *DLX6-AS1* with *PRINS* to diagnose DR in males.

We further explored whether plasma exosome lncRNAs could predict a DR diagnosis. The areas under the curves (AUCs) for *DLX6-AS1* [sensitivity: 0.500, specificity: 0.790, 95% confidence interval (CI): 0.562–0.754, *P* = 0.002], *PRINS* (sensitivity: 0.774, specificity: 0.661, 95% CI: 0.722–0.873, *P* < 0.001) and *FAM190A-3*(sensitivity:0.403, specificity:0.790, 95% CI: 0.503-0.702, *P* = 0.048)were 0.658, 0.798 and 0.048, respectively, and all were significant, indicating that *DLX6-AS1* and *PRINS* have a certain ability to predict DR. However, *ACY1* and *ARHGAP* were not found to be significant predictors of DR diagnosis (**Figure 1A**). The AUC for the model combining *DLX6-AS1* with *PRINS* yielded an AUC of 0.813 (sensitivity: 0.710, specificity: 0.758, 95% CI: 0.740–0.886, *P* < 0.001), which was higher than the values for the single-factor models (**Figure 1B**). In a subgroup analysis by sex, the AUC of *DLX6-AS1* in males was 0.697 (0.580–0.815). *PRINS* yielded AUCs of 0.792 (0.695–0.889) in males and 0.818 (0.695–0.940) in females. *FAM190A-3*, *ACY1*, and *ARHGAP* were not found to have significant diagnostic value for DR in either men or women (**Figures 1C, D**). In male patients with DR, the model combining *DLX6-AS1* with *PRINS* yielded an AUC of 0.860 (0.780-0.940), which was larger than the value produced by either single-factor model (**Figure 1E**) (**Supplementary Table 6**). The predictive effect of lncRNAs on DR might be achieved by regulating genes encoding cell membrane components and intercellular signal transduction. Because we found that a total of 37 cis-regulated target genes were predicted to have some similarity in GO function through the differential DR-related lncRNAs in our and previous studies, including lncRNAs *DLX6-AS1*, *PRINS*, *FAM190A-3*, *MALAT1*, *ANRIL*, *RNCR3*, *MIAT*, and *Sox2OT* (**Supplementary Figure 3**).

## 4 Discussion

We found that some abnormalities in biochemical indicators, such as the fasting blood glucose concentration, 2-h C-peptide concentration, and HbA1c level, and plasma exosomal lncRNA expression, such as *DLX6-AS1*, *PRINS*, and *FAM190A-3* might be associated with the pathological changes of DR. Of particular note is the the combination of exosomal *DLX6-AS1* and *PRINS* had good diagnostic value for DR in the general population and males.

Early screening for DR requires close attention to indicators associated with T2DM. Compared with patients with T2DM in our study, DR patients exhibited increases in the fasting blood glucose concentration and HbA1c level and decreases in the 2-h insulin and fasting and 2-h C-peptide concentrations. Accordingly, the fasting blood glucose concentration and HbA1c level might be risk factors for DR, while the 2-h C-peptide concentration might be a protective factor. A meta-analysis showed that patients with T2DM patients who had high fasting blood glucose concentrations and HbA_1c_ levels faced significant increases in the risk of developing DR (33% and 15%, respectively) (21). Retinal blood vessels are sensitive to the blood glucose status. During early hyperglycemia, retinal blood vessels progressively dilate, and the blood flow changes autonomously to increase retinal metabolism and maintain retinal function (22). During long-term hyperglycemia, the proliferation of retinal endothelial cells and thickening of the retinal basement membrane eventually damage the blood–retinal barrier. In addition, a reduction in pericytes causes local extravasation of the capillary wall and eventual microaneurysm formation (23). The decreased insulin and C-peptide concentrations observed in patients with DR imply that impairment of islet cell function associated with T2DM increases the risk of developing DR (24). Accordingly, patients with T2DM who have poor long-term blood glucose control need regular fundus examinations to ensure the early diagnosis and treatment of DR.

Our further analysis revealed significant decrease in total bilirubin and uric acid in the DR group relative to the T2DM group, consistent with the results of previous studies (25). This change might be related to the role of bilirubin in oxidative stress (26) and inflammatory responses (27, 28), which are important risk factors for the development of DR. Studies have not yet determined whether changes in the uric acid concentration are associated with DR (29), and this potential relationship needs to be confirmed by a large-sample cohort study. Accordingly, we should consider continuous changes in the total bilirubin or uric acid concentrations in patients with T2DM when confirming the occurrence of DR.

An exosome is an extracellular vesicle that contains a variety of bioactive molecules. After an exosome is taken up by a target cell, exosomal RNAs are translated and expressed as proteins; accordingly, exosomes play a role in intercellular communication and mediate physiological or pathological processes (30). Studies have shown that plasma samples from diabetic patients contain large numbers of exosomes (31), the transport of which promotes the production of angiogenic factors. Therefore, exosomes may play an important role in the occurrence and development of DR. Plasma exosomes isolated from patients with DR were found to be enriched for many components that can regulate inflammatory responses and angiogenesis (32), such as activated T cells that express and secrete factors, vascular endothelial growth factor, and angiopoietin 2. Exosomes can also increase cell permeability through pericyte detachment and endothelial cell migration, resulting in the formation of blood vessel-like structures (33). Serum exosomes are rich in arginase 1, which is taken up by endothelial cells, where it inhibits the formation of nitric oxide and mediates vascular endothelial cell dysfunction (34). In the current study, we identified associations between the occurrence of DR and several plasma exosomal lncRNAs, namely *DLX6-AS1*, *PRINS*, and *FAM190A-3*. In particular, a diagnostic model that combined *DLX6-AS1* and *PRINS* was shown to have good predictive value for DR. Our findings provide new ideas for further research on the function of exosomes in the occurrence and development of DR.

The lncRNA *DLX6-AS1* is located in chromosomal region 7q21.3 (chromosome 7: 96968515-97014065). This natural antisense RNA can regulate the corresponding sense mRNA and exert corresponding biological functions. Research on the function of *DLX6-AS1* has focused mainly on its role in malignancy (35). However, some studies have found that *DLX6-AS1* is related to the occurrence of diabetic nephropathy (36), as its expression is upregulated in patients with diabetes; in this context, *DLX6-AS1* inhibits the expression and function of miRNA-346 and eventually causes kidney damage. We found that *DLX6-AS1* was highly expressed in DR, and further logistic regression analysis indicated that this lncRNA was both associated with DR and a risk factor for DR. The functional molecular mechanism of *DLX6-AS1* includes the MAPK signaling pathway (37), indicating that *DLX6-AS1* might play a role in DR pathogenesis by regulating the p38–MAPK pathway, which was proved to be related to the occurrence and development of DR, and by regulating endothelial cell function.

*PRINS* is located in chromosomal region 10p12.31 and is known to be closely related to psoriasis. In the pathogenesis of psoriasis, *PRINS* inhibits the expression of cellular inflammatory factors [e.g., interleukin (IL)-1α, IL-1β, IL-6, IL-8, and tumor necrosis factor-α) and thus affects inflammatory responses (38). *PRINS* may contribute to diabetic complications by regulating the expression of Smad7, an important transduction molecule in transforming growth factor (TGF)-β/Smad signaling pathways (39). Smad7 can inhibit TGF-β-mediated signaling by inhibiting the phosphorylation of R-Smad. TGF-β is a cytokine that regulates gene expression, cell proliferation and differentiation, and apoptosis, and research has confirmed its wide involvement in DR pathogenesis (40). In our study, we observed a significant decrease in *PRINS* expression, which might have led to a decrease in Smad7 expression and, consequently, a decrease in its protective effect on the retina.

*FAM190A-3*, which is also known as lnc-*CCSER1-3*, is located in chromosomal region hg38:chr4:91659023-91660116. In our total study population, *FAM190A-3* was expressed at higher levels in the T2DM group than in the DR group. However, in the subgroup analysis by sex, we found no significant difference between the T2DM and DR groups. In a further logistic regression analysis, we observed no association of *FAM190A-3* with DR after adjusting for relevant variables. Therefore, the role of *FAM190A-3* in the pathogenesis of DR remains to be confirmed.

We have found that in differential lncRNA targeting genes and GO function predictions, DR-related lncRNAs play an important role in cells, especially cell membrane components and intercellular signaling. In the future, we can construct differential lncRNA-mRNA pairwise interaction gene regulatory networks, explore the correlation of target genes expression and the effect of lncRNA-mRNA on target genes, further verify the function of target genes, and enrich the mechanism of DR pathogenesis at the exosome level. In addition, fractionation of exosome sub-population will help to eliminate the effect of heterogeneity. Single exosome surface membrane proteome detection and protein fingerprint characteristic can be carried out, or affinity purification can be performed using exosome capture kits with different antibodies to obtain classification of exosome sub-population (41). Further observation of the heterogeneity of lncRNA content and the potential roles of different exosome sub-population will deepen our understanding of the pathological mechanism of exosomes and DR.

This study is the first to systematically explore the association of exosomal lncRNA expression with DR pathogenesis in patients with T2DM. We have confirmed that some exosomal lncRNAs are associated with DR pathogenesis and have predictive value for DR diagnosis. However, this study has some limitations. First, it was a cross-sectional case-control study, and therefore, we cannot clarify causal associations between exosomal lncRNAs and DR. Secondly, the participants in this study were all of Han Chinese ethnicity and hospital inpatients in a single region. The single-center nature of the data source may have introduced selection bias. Finally, the population of patients with DR in our study included few patients with proliferative-stage retinopathy, and thus, this study did not explore differences in the exosomal lncRNAs between patients with different stages of DR. We have found that there are differences in exosome expression between DR classification. It is necessary to expand the sample source, explore the exosome mechanism of different DR classification through cohort studies in the future.

In conclusion, we have identified a close relationship between exosomes and the occurrence and development of DR. Furthermore, some exosomal lncRNAs, such as *DLX6-AS1* and *PRINS*, were found to have value for the diagnosis and pathogenesis of DR. Our findings elucidate a new method for the early diagnosis of DR, which could effectively reduce the burden associated with this disorder.

## Data availability statement

The data presented in the study are deposited in the GEO repository, accession number GSE212594.

## Ethics statement

The studies involving human participants were reviewed and approved by the Ethics Committee of Ningbo First Hospital (approval number: 2017-R048). The patients/participants provided their written informed consent to participate in this study. Written informed consent was obtained from the individual(s) for the publication of any potentially identifiable images or data included in this article.

## Author contributions

BH and TZ conceived the ideas for the study, and provided overall guidance. MX and LL (5th author) provided the demographic and clinical characteristics data, and conducted the sample collection. QYe (1st author), ZS, and QYa (6th author) performed exosome identification and real-time quantitative PCR. LL (1st author) and TZ conducted data analysis, figure and chart production, and TZ completed the article writing. All authors contributed to the article and approved the submitted version.

## Funding

The study is supported by grants from by the Ningbo Health Branding Subject Fund (PPXK2018-01).

## Acknowledgments

We would like to thank Guangzhou Geneseed Biotechnology Co., Ltd. (Guangzhou, China) for providing sequencing services and helpful discussions pertaining to the sequencing and data analysis.

## Conflict of interest

The authors declare that the research was conducted in the absence of any commercial or financial relationships that could be construed as a potential conflict of interest.

## Publisher’s note

All claims expressed in this article are solely those of the authors and do not necessarily represent those of their affiliated organizations, or those of the publisher, the editors and the reviewers. Any product that may be evaluated in this article, or claim that may be made by its manufacturer, is not guaranteed or endorsed by the publisher.

## Abbreviations

lncRNA, long noncoding RNA; DR, diabetic retinopathy; T2DM, type 2 diabetes mellitus; PCR, polymerase chain reaction; ROC, receiver operating characteristic; GO, GeneOntology; HbA_1c_, glycosylated hemoglobin level; AUC, area under the curve; KEGG, Kyoto Encyclopedia of Genes and Genomes; *DLX6-AS1*, distal-less homeobox 6 antisense 1; *PRINS*, psoriasis-susceptibility-related RNA gene induced by stress; *FAM190A-3*, family with sequence similarity 190, member A3; *ACY1*, aminoacylase-1, *ARHGAP*, Rho GTPase activating protein; TGF, transforming growth factor.
