# Supplementary material for: Association between lncRNAs in plasma exosomes and diabetic retinopathy
Source: Front Endocrinol (Lausanne). 2022 Sep 14;13:987488. doi: 10.3389/fendo.2022.987488 (PMC9519175; doi:10.3389/fendo.2022.987488)
Supplement: Supplementary file 1 [file DataSheet_1.docx]

ssSupplementary Material

# Supplementary Figures and Tables

## Supplementary Figures


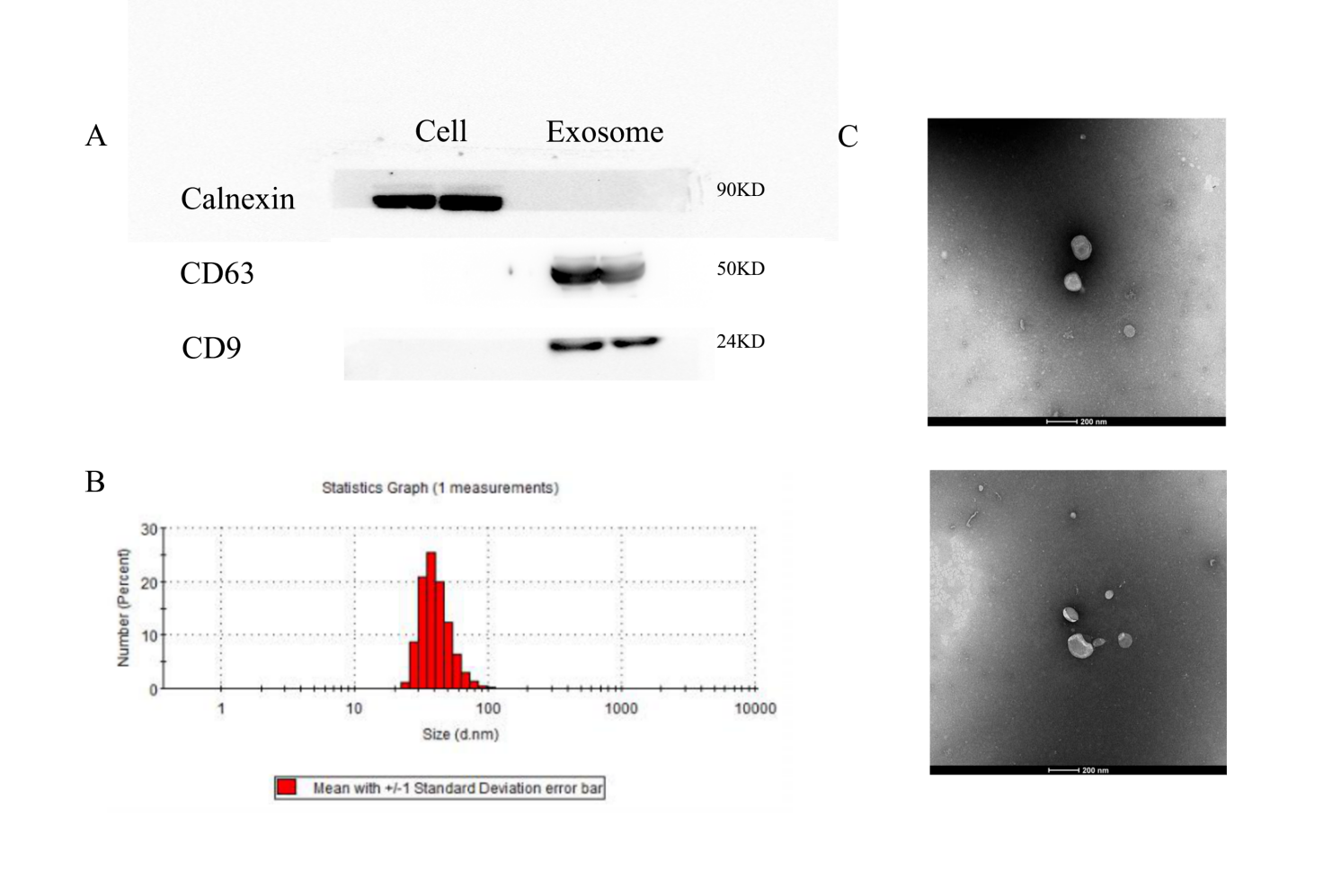


**Supplementary Figure 1.** **The results of exosome identification.** (A) The expression of related marker proteins Calnexin, CD63, and CD9 molecules in plasma and plasma exosomes. (B) Plasma exosome particle size. (C)Plasma exosomes identified by transmission electron microscopy. Bars, 200nm.


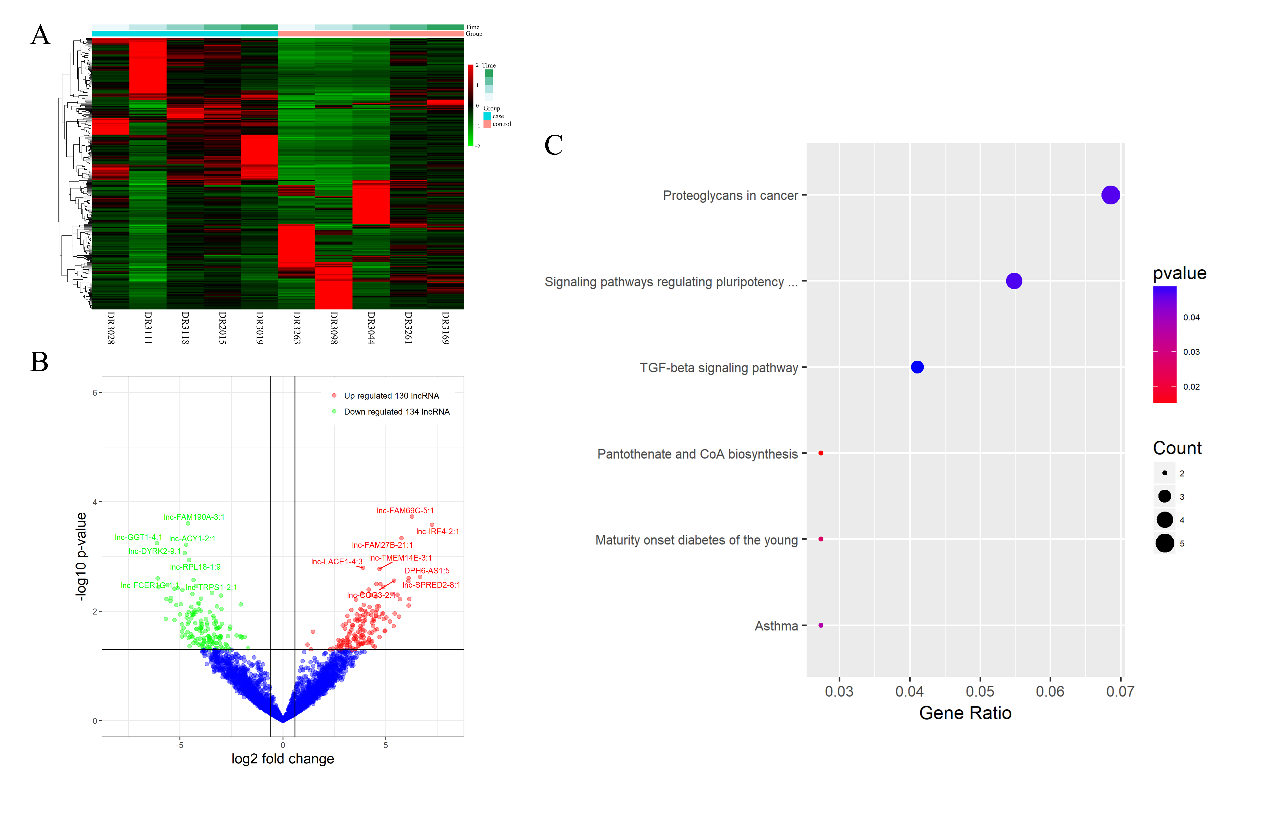


**Supplementary Figure 2**. **Comparison of significantly differentially expressed lncRNA genes between DR and T2DM groups.** (A) Heatmap between the two groups. Each column represents a sample, and each row represents a gene, with up-regulated expression in red and down-regulated expression in green. (B) Volcano map between the two groups. Each point represents a gene. The x-axis represents the fold of gene expression difference ≥ 1.5 as the standard, log2 (fold change) is the value after de-logarithm of the fold of gene expression difference, and the y-axis represents the significance of gene expression difference with -log10 conversion. Up-regulated genes expresses in red and down-regulated genes expresses in green. (C) Dot plot of Kyoto Encyclopedia of Genes and Genomes pathway enrichment analysis in differentially expressed genes. Abbreviations: lncRNA, long noncoding RNA; DR, diabetic retinopathy; T2DM, type 2 diabetes mellitus.


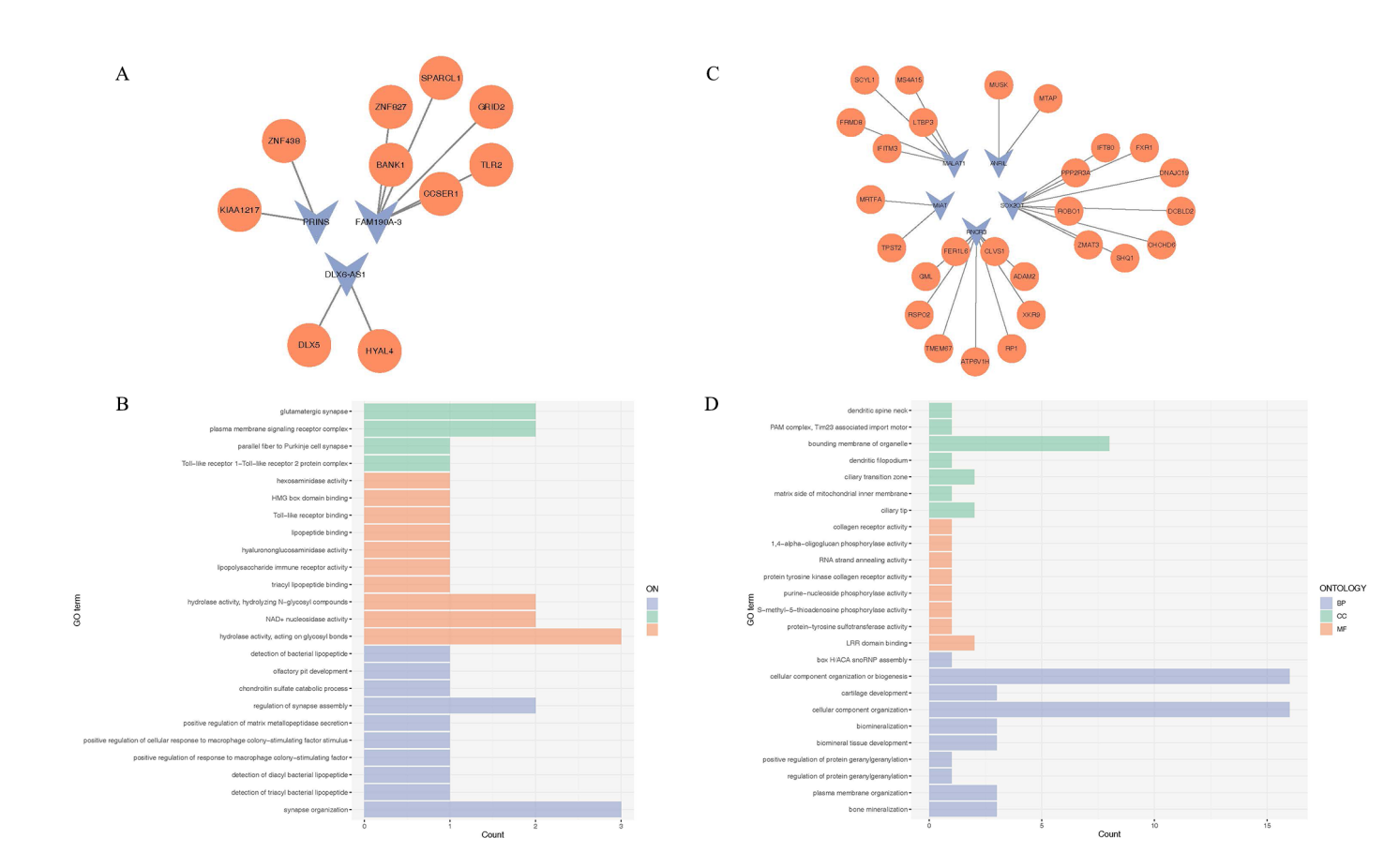


**Supplementary Figure 3. Potential cis-regulated target genes of all identified lncRNAs and their GO enrichment pathways.** （A）Potential cis-regulated target genes and （B）GO enrichment pathways of lncRNAs *DLX6-AS1, PRINS,* and *FAM190A-3.*（C）Potential cis-regulated target genes and （D）GO enrichment pathways of lncRNAs *MALAT1*, *ANRIL*, *RNCR3*, *MIAT*, and *Sox2OT.* Abbreviations: lncRNA, long noncoding RNA; GO, GeneOntology.

## Supplementary Tables

**Supplementary Table 1. Diabetic Retinopathy Questionnaire.**

| Version number V1.0 Version time 2018.01 | |
| --- | --- |
| **A. Basic situation** | A1. Name: _________________, Contact number ________________ |
|  | A2. Sex: 1.Male 2.Female |
|  | A3. Ethnic: 1.Han 2.Others, please specify ____________ |
|  | A4. Height: ____________cm |
|  | A5. Weight: ____________kg |
|  | A6. Systolic blood pressure (SBP): _________mmHg |
|  | A7. Diastolic blood pressure (DBP): _________mmHg |
|  | A8. Waistline: ____________cm |
|  | A9. Hip circumference: ____________cm |
|  | A10. Age: ____________ Date of birth: ___________ |
|  | A11. Education: ____________  1.Illiteracy 2.Elementary school 3.Junior high school 4.High school/secondary school 5.Undergraduate/college 6.Graduate and above |
|  | A12. Marital status: ____________  1.Unmarried 2.Married 3.Remarried 4.Divorced 5.Widowed |
|  | A13. Occupation: ___________  1. Worker 2.Peasant 3.Soldier 4.Administrative cadre 5.Technical staff  6. Medical staff 7.Teacher 8.Finance 9.Business service staff 10.Housewife  11. Retired and retired 12.Unemployed 13.Student  Physical labor intensity: ___________  1. Light physical labor 2. Medium physical labor 3. Heavy physical labor |
| **B. Smoking and drinking** | B1. Do you currently smoke (including cigarettes, hand-rolled cigarettes, pipes, hookahs, chewing tobacco, snuff, cigars, etc.)?  1. Yes 2. I used to smoke, but now I have quit...B3 3. No...B3 |
|  | B2. How much do you usually take in a day? ________branch |
|  | B3. Do you drink alcohol? Drinking: drinking at least once a week for more than half a year. Abstinence refers to not drinking for more than half a year.  1. Yes 2.No... C1 3. I have quit drinking... C1 |
| **C. Disease** | C1. Do you have a family history of diabetes? ___________  1. Yes 2. No |
|  | C2. History of diabetes for __________ years |
|  | C3. Do you have any of the following diabetes complications?  1.diabetic retinopathy 2.diabetic nephropathy 3.diabetic neuropathy 4.diabetic foot |
|  | C4. Do you have other diseases besides diabetes? specifically is:  ________________, ________________, ________________, ________________, ________________, ________________, ________________, ________________. (Note: If you have liver disease, please indicate) |
|  | C5. The grade of diabetic retinopathy: _______________  (Note: This is to be filled in by the ophthalmologist)  1. No obvious retinopathy 2. Non-proliferative retinopathy 3.Proliferative retinopathy |
| **D. Biochemical indicators** | D1. Fasting blood glucose (FBG): _________mmol/L |
|  | D2. 2-hour postprandial blood glucose (2h PBG): _________mmol/L |
|  | D3. Glycosylated hemoglobin (HbA_1c_): _________% |
|  | D4. Total cholesterol (TC): _________mmol/L |
|  | D5. Triglyceride (TG): _________mmol/L |
|  | D6. High density lipoprotein (HDL): _________mmol/L |
|  | D7. Low density lipoprotein (LDL): _________mmol/L |
|  | D8. Total bilirubin (TBil): _________μmol/L |
|  | D9. Hemoglobin (Hb): _________g/L |
|  | D10. Uric acid (UA): _________mmol/L |
|  | D11. Homocysteine (Hcy): _________ μmol/L |
|  | D12. Free Fatty Acids (FFA): _________ mmol/L |
|  | D13. Fasting C-peptide (C-P): _________ nmol/mL |
|  | D14. 2 hours postprandial C-peptide (2h C-P ): _________ nmol/mL |
|  | D15. Fasting insulin (INS): _________ mmol/L |
|  | D16. 2h insulin (2h INS): _________ mmol/L |
| 1 Questionnaire has been completed  2 Part of questionnaire has been completed  3 Refused to be investigated | |
| Investigator signature: ________________________ | |

**Supplementary Table 2**. **The primer sequences for lncRNA and internal references.**

| **Gene** | **Forward primer (5'‑3')** | **Reverse primer (5'‑3')** |
| --- | --- | --- |
| *DLX6-AS1* | CCACCCACTGAGAGAAGAGG | CCTCCAAGCAATTGTCCAGT |
| *PRINS* | TCCACATCCGGATTTACCTAAAC | CTGACGCACCCCTGACAGTCAG |
| *FAM190A* | AGTGGTTGACGGACAGTGAG | TCCTCCTGTTCTGTGCTCC |
| *ACY1* | GGGTCGCACCAGTCCTTCT | GCCCCAGCTTCAGTTCCAG |
| *ARHGAP21* | GATGTTCTTCAACTCCACCCT | AATGACTGACTTCTTGCCTCC |
| *18S* | CCTGGATACCGCAGCTAGGA | GCGGCGCAATACGAATGCCCC |

**Supplementary Table 3**. **The differences in expression levels of exosome lncRNA between DR and T2DM groups with or without fatty liver disease.**

|  | **DR** | **T2DM** | ***t*** | ***P*** |
| --- | --- | --- | --- | --- |
| ***DLX6-AS1*** |  |  |  |  |
| Fatty liver disease | 1.34±0.76 | 1.09±0.57 | -1.800 | 0.075 |
| Non- fatty liver disease | 1.37±0.67 | 0.91±0.27 | -2.502 | 0.019 |
| ***PRINS*** |  |  |  |  |
| Fatty liver disease | 0.98±0.15 | 1.21±0.26 | 5.039 | <0.001 |
| Non- fatty liver disease | 1.03±0.08 | 1.19±0.23 | 2.041 | 0.051 |
| ***FAM190A-3*** |  |  |  |  |
| Fatty liver disease | 1.05±0.07 | 1.09±0.13 | 2.101 | 0.038 |
| Non- fatty liver disease | 1.03±0.34 | 1.11±0.13 | 0.899 | 0.377 |
| ***ACY1*** |  |  |  |  |
| Fatty liver disease | 1.30±0.58 | 1.45±0.82 | 0.943 | 0.348 |
| Non- fatty liver disease | 1.23±0.26 | 1.14±0.19 | -0.956 | 0.349 |
| ***ARHGAP*** |  |  |  |  |
| Fatty liver disease | 1.29±0.45 | 1.28±0.38 | -0.123 | 0.902 |
| Non- fatty liver disease | 1.40±0.80 | 1.30±0.34 | -0.423 | 0.676 |

Abbreviations: lncRNA, long noncoding RNA; DR, Diabetic retinopathy; T2DM, Type 2 diabetes mellitus.

**Supplementary Table 4.** **Correlation analysis of exosome lncRNA and biochemical indicator.**

|  | ***DLX6-AS1*** | | ***PRINS*** | | ***FAM190A-3*** | |
| --- | --- | --- | --- | --- | --- | --- |
|  | *r* | *P* | *r* | *P* | *r* | *P* |
| Total bilirubin (μmol/L) | 0.107 | 0.317 | 0.222 | 0.036 | -0.157 | 0.141 |
| Uric acid (mmol/L) | -0.166 | 0.127 | 0.021 | 0.845 | 0.006 | 0.959 |
| HbA_1c_ (%) | 0.077 | 0.514 | -0.247 | 0.034 | -0.069 | 0.561 |
| Fasting blood glucose (mmol/L) | 0.183 | 0.148 | -0.204 | 0.105 | -0.013 | 0.917 |
| 2h blood glucose (mmol/L) | 0.146 | 0.252 | 0.054 | 0.672 | -0.229 | 0.070 |
| Fasting insulin (mmol/L) | 0.067 | 0.603 | 0.188 | 0.141 | 0.054 | 0.676 |
| 2h insulin (mmol/L) | 0.017 | 0.895 | 0.293 | 0.017 | -0.103 | 0.409 |
| Fasting C-peptide (nmol/mL) | -0.091 | 0.476 | 0.136 | 0.283 | 0.165 | 0.192 |
| 2h C-peptide (nmol/mL) | -0.132 | 0.291 | 0.265 | 0.032 | -0.002 | 0.990 |

Abbreviations: lncRNA, long noncoding RNA; HbA_1c_, Glycosylated hemoglobin.

**Supplementary Table 5. Multivariate logistic regression analysis in different subgroups.**

|  | ***DLX-6AS1*** | ***P*_interaction_** | ***PRINs*** | ***P*_interaction_** | ***FAM190A-3*** | ***P*_interaction_** |
| --- | --- | --- | --- | --- | --- | --- |
| **Sex** |  |  |  |  |  |  |
| Males | 6.688(2.135-20.952) | 0.097 | 0.151(0.048-0.474) | 0.803 | 0.246(0.075-0.799) | 0.394 |
| Females | 1.369(0.284-6.610) |  | 0.047(0.006-0.379) |  | 0.371(0.074-1.844) |  |
| **Age (years)** |  |  |  |  |  |  |
| ≤60 | 2.591(0.943-7.121) | 0.167 | 0.096(0.028-0.332) | 0.502 | 0.564(0.171-1.863) | 0.216 |
| ＞60 | 10.061(1.473-68.719) |  | 0.130(0.019-0.881) |  | 0.077(0.011-0.522) |  |
| **Smoker** |  |  |  |  |  |  |
| Yes | 8.048(1.259-51.457) | 0.202 | 0.049(0.006-0.394) | 0.914 | 0.181(0.029-1.151) | 0.497 |
| No | 2.716(0.869-8.482) |  | 0.146(0.042-0.514) |  | 0.383(0.112-1.312) |  |
| **Alcohol consumption** |  |  |  |  |  |  |
| Yes | 12.942(1.216-137.774) | 0.273 | 0.093(0.009-0.913) | 0.336 | 0.488(0.058-4.090) | 0.502 |
| No | 2.398(0.907-6.342) |  | 0.150(0.052-0.436) |  | 0.228(0.072-0.717) |  |

**Supplementary Table 6. The receiver operating characteristic curve analysis of the diagnostic value of exosome lncRNA for DR.**

|  | **Sensitivity** | **Specificity** | **AUC** | **95% CI** | ***P*** |
| --- | --- | --- | --- | --- | --- |
| ***DLX6-AS1*** | 0.500 | 0.790 | 0.658 | 0.562-0.754 | 0.002 |
| Males | 0.641 | 0.718 | 0.697 | 0.580-0.815 | 0.003 |
| Females | 0.739 | 0.522 | 0.597 | 0.430-0.764 | 0.258 |
| ***PRINS*** | 0.774 | 0.661 | 0.798 | 0.722-0.873 | <0.001 |
| Males | 0.538 | 0.923 | 0.792 | 0.695-0.889 | <0.001 |
| Females | 0.739 | 0.783 | 0.818 | 0.695-0.940 | <0.001 |
| ***FAM190A-3*** | 0.403 | 0.790 | 0.603 | 0.503-0.702 | 0.048 |
| Males | 0.487 | 0.718 | 0.601 | 0.475-0.727 | 0.125 |
| Females | 0.348 | 0.870 | 0.605 | 0.441-0.769 | 0.223 |
| ***ACY1*** | 0.491 | 0.627 | 0.516 | 0.405-0.626 | 0.778 |
| Males | 0.633 | 0.472 | 0.531 | 0.389-0.672 | 0.671 |
| Females | 0.632 | 0.667 | 0.608 | 0.426-0.789 | 0.224 |
| ***ARHGAP*** | 0.636 | 0.453 | 0.507 | 0.397-0.617 | 0.900 |
| Males | 0.629 | 0.529 | 0.571 | 0.435-0.706 | 0.313 |
| Females | 0.474 | 0.900 | 0.611 | 0.422-0.799 | 0.238 |
| ***DLX6-AS1+ PRINS*** | 0.710 | 0.758 | 0.813 | 0.740-0.886 | <0.001 |
| Males | 0.770 | 0.795 | 0.860 | 0.780-0.940 | <0.001 |

Abbreviations: lncRNA, long noncoding RNA; DR, Diabetic retinopathy; AUC, Area under curve; CI, confidence interval.
